# Numerical simulation of oscillatory oblique stagnation point flow of a magneto micropolar nanofluid

**DOI:** 10.1039/c8ra09698h

**Published:** 2019-02-06

**Authors:** Muhammad Adil Sadiq, Arif Ullah Khan, S. Saleem, S. Nadeem

**Affiliations:** Department of Mathematics, DCC-KFUPM Box 5084 Dhahran 31261 Saudi Arabia; Department of Mathematics, Quaid-i-Azam University 45320 Islamabad 44000 Pakistan aukhan@math.qau.edu.pk; Department of Mathematics, College of Sciences, King Khalid University Abha 61413 Saudi Arabia saakhtar@kku.edu.sa; Department of Mathematics, University of Science and Technology Bannu KPK Pakistan

## Abstract

The particular inquiry is made to envision the characteristics of magneto-hydrodynamic oscillatory oblique stagnation point flow of micropolar nanofluid. The applied magnetic field is assumed parallel towards isolating streamline. A relative investigation is executed for copper and alumina nanoparticles while seeing water type base fluid. To be more specific, in the presence of both weak and strong concentration, the physical situation of micropolar fluid is mathematically modeled in terms of differential equations. The transformed mixed system is finally elucidated by midpoint method with the Richardson extrapolation development and shooting mechanism with fifth order R–K Fehlberg technique. The impact of governing parameters are shown and explored graphically. The obtained results are compared with existing published literature. Moreover, it is found that the magnetic susceptibility of nanofluids shows provoking nature towards copper as compared to Alumina. Also it is perceived that Cu–water shows higher wall shear stress and heat transfer rate than Al_2_O_3_–water. Additional, the thickness of momentum boundary layer is thin for weak concentration as related to strong concentration.

## Abstract 2

The particular inquiry is made to envision the characteristics of magneto-hydrodynamic oscillatory oblique stagnation point flow of micropolar nanofluid.

## Graphical abstract

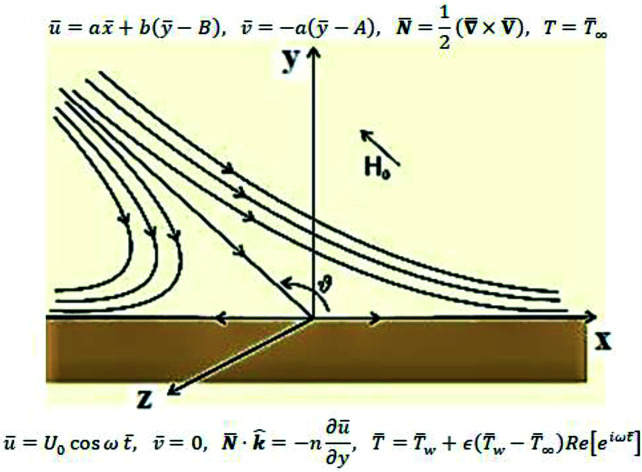

## 1. Introduction

The study of nanofluids claims remarkable practical applications in investigational and modern disciplines. The disclosure of nanoparticles has enhanced the proficiency and decreased the budget of cooling and warming structures. As anyone might expect, analysts additionally notice the subsequent information: the nanoparticles enhance both the thermal conductivity and apparent viscosity significantly of the nanofluid. Thus, additional propelling power is needed to retain the nanofluid flow over the device. The reduction in pumping cost for a cooling system because of small size particles was probed by Choi.^1^ Murshed *et al.*^2^ observed that the operative thermal conductivity and viciousness of nanofluids considerably enhanced by particle volume fraction. The variation of thermal conductivity for nanofluids largely depends on the shape, size, and material of which nanoparticles are made. The effectiveness of nanoparticles is not limited towards coolants. Scientists and researchers have noticed a few other successful employments of particles examined in ref. 3. Nanofluids are presently being created for therapeutic applications, including cancer treatment and safe surgery by cooling. The blockage of UV rays can be achieved by using nanoparticles (zinc oxide) into plastic packaging and also offer an anti-bacterial shield. This helps to rectify the strength and stability of the plastic sheets. A recent application of nanofluid flow is nano-drug delivery. Pak and Cho^4^ deliberated the characteristics of heat flux in the fluid by adding metallic oxide particles (nanometer-sized) to it. This attempt reports that the inciting nature of heat transfer is actually because of the alteration in thermal conductivity of the metallic particles and common fluid. Aftab *et al.*^5^ investigated the nanoconfined phase change materials for thermal energy applications. Far along^6–12^ noticed that the nanoparticles are the source of variations in heat transfer. It is important to note that during these attempts uniform distributions of nanoparticles are assumed throughout the flow regime. Buongiorno^13^ identified such elements entertained velocity slips towards base fluid molecules are unable to maintain uniform concentration throughout the flow regime. The mechanism of seven-slip was proposed by him who can play an active role in the heat transfer enhancement. Further, he declared that both thermal and Brownian diffusion is most active slip mechanisms. Sheikholeslami and Ganji^14^ scrutinized the nanofluid flow together with heat transfer by means of corresponding plates squeezing. They established the direct relation of Nusselt number with nanoparticle volume fraction, Eckert number and squeeze number towards separated plates. Recently, Khan *et al.*^15^ explore the magneto-hydrodynamic flow under the region of oblique stagnation point with slip effect for water-based nanofluid containing, and (as nanoparticles). They found that –water is the source of enrichment of heat transfer at the sheet followed by –water and –water. Nadeem *et al.*^16^ examined the model-based study of SWCNT and MWCNT thermal conductivities effect on the heat transfer due to the oscillating wall conditions. Combined effects of viscous dissipation and Joule heating on MHD Sisko nanofluid over a stretching cylinder was deliberated by Hussain *et al.*^17^ Sheikholeslami *et al.*^18^ described the flow of CuO nanoparticles with radiation and discharging rate.

An analysis of micropolar liquids has captivated the devotion of investigators and experts in the field of fluid science. Such consideration is due to fact that the conventional Newtonian fluids cannot depict the complete description of fluid flow in various biological and industrial applications. For polar fluids, a distinct and special kind of microstructure material claims stress tensor which should be non-symmetric. Basically, in terms of the physical frame, it depicts those materials which consist of situated molecules (arbitrarily) cast out in a viscous liquid. The variations in couple stress, body couples, micro-rotational and disclose micro-inertial are supported by polar fluids. In general, the majority of the physiological liquids treated as polar fluid like suspensions of rigid or deformable particles in the viscous fluid, plasma, and cervical. In short, Eringen^19^ was the earliest to mention the theory of micropolar fluids. After his study, micropolar fluids have been recognized widely by researchers because of numerous engineering and industrial applications. To mention just a few, cervical flows, contaminated and clean engine lubricants, colloids and polymeric suspensions, thrust bearing technologies and radial diffusion paint rheology.

Micropolar fluids do act together closely with nanofluids as micropolar fluids are fluids with microstructure and nanofluid are colloidal suspension of metallic or non-metallic nanosize particles in the base fluid. Physically micropolar fluids symbolize fluids comprising of rigid, randomly oriented (or spherical) particles suspended in a viscous medium. These particles may be of nano-size, which actually makes the micropolar fluids to behave as nanofluids. Physical examples of micropolar fluids can be seen in ferrofluids, blood flows, bubbly liquids, liquid crystals, and so on, all of them containing intrinsic polarities. It is important to note that some of these micropolar fluids do behave as nanofluid with heat transfer enhancement characteristics due to the presence of nanosize particles apart from their intrinsic polarities. The addition of nanoparticles in a micropolar fluid, make the mixture more complex as compare to conventional nanofluids and offer investigators with a new dimension to explore the fluid flow characteristics.

The fluid (cerebrospinal) motion in the brain was identified by Power^20^ and he has shown that the Cerebrospinal fluid is adequately modeled through micropolar fluids. Lukaszewicz^21^ explained in his book about the physical aspects of micropolar fluids regarding practical applications. Das^22^ examined the flow characteristics of heat-mass transfer in the micropolar fluid over an inclined sheet along with both chemical reaction and thermophoresis effects. Double diffusive unsteady convective micropolar flow past a vertical porous plate moving through binary mixture using modified Boussinesq approximation was discussed by Animansaun.^23^ Recently, the impact of temperature dependent viscosity on the micropolar fluid flow by way of two nanofluids was taken by Nadeem *et al.*^24^ They found that both micro rotation viscosity and micro inertia density are a function of temperature dependent dynamic viscosity. More recently, Gina Nov *et al.*^25^ scrutinized the properties of micropolar fluid flow in a wavy differentially heated cavity with natural convection effect. Whereas, the impression of magnetic field on micropolar fluid flow along a vertical channel was explored by Borrelli *et al.*^26^ Some important literature which enhances the features of micropolar fluids is given in ref. 27–29. Takhar *et al.^29^* examined MHD flow over a moving plate in a rotating fluid with the magnetic field, Hall currents, and free stream velocity. MHD stagnation point flow with flexible features was investigated by Khan *et al.^30^* Genuinely, in the manufacturing production of polymer fluids, colloidal solutions and the fluid having minor additives; there is frequently a point where the local velocity of the fluid owns symmetric stress tensor and micro-rotation of particles is nil. Some of the important studies about stagnation point flow are given in ref. 31–34.

**Fig. 1:**
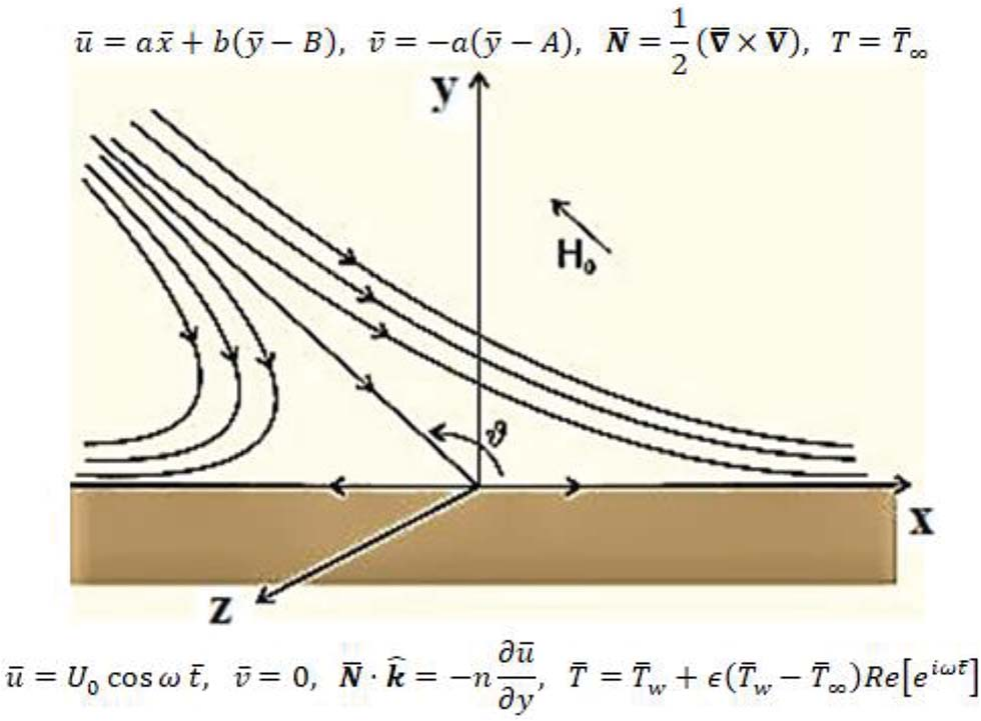
Flow description of the problem.

The review of the above-mentioned literature reflects that as yet, the impact of magnetic field on oblique stagnation point flow of micropolar nanofluid with the manifestation of copper and alumina nanoparticles is not been addressed on an oscillating surface. So, in this article, the influence of nanoparticles is added by activating nanoparticles viscosities and thermal conductivity effective model. The problem is of more importance because, micropolar fluids do interact closely with nanofluids since micropolar fluids are fluids with microstructure and nanofluid are colloidal suspension of metallic or non-metallic nanosize particles in the base fluid. Physically speaking, micropolar fluids represent fluids consisting of rigid, randomly oriented (or spherical) particles suspended in a viscous medium. These particles may be of nano-size, which indeed makes the micropolar fluids to behave as nanofluids. Physical examples of micropolar fluids can be seen in ferrofluids, blood flows, bubbly liquids, liquid crystals, and so on, all of them containing intrinsic polarities. It is important to note that some of these micropolar fluids do behave as nanofluid with heat transfer enhancement characteristics due to the presence of nanosize particles apart from their intrinsic polarities. The physical flow illustration of the problem is mathematically modeled in terms of partial differential equations. A suitable similarity transformation is used to attain ordinary differential equations. The converted differential equations (coupled system) are ultimately unraveled by using BVP solution method and shooting scheme along with fifth order R–K Fehlberg algorithm. A brief parametric analysis is executed to inspect the effect logs of involved physical parameters on dimensionless velocity and temperature by way of graphical attitudes. Further, the tabular structure is also design to analyze the variation of some physical quantities namely, velocity and temperature gradients adjacent to the flat surface. The obtained results are compared with existing published literature. An excellent match has been found which yields the validity of the current analysis. In the last it is mentioned here that the addition of nanoparticles in a micropolar fluid, make the mixture more complex as compare to conventional nanofluids and provide researchers with a new dimension to explore the fluid flow characteristics (Fig. 1).

## 2. Problem description and governing equations

Consider the problem of stagnation point flow of an electrically conducting micropolar nanofluid over an oscillatory surface with velocity *U*_0_ cos *ωt̄*. The fluid impinges obliquely to the oscillatory surface *ȳ* = 0. By neglecting external mechanical body force and body couple the flow rheological equations becomes1
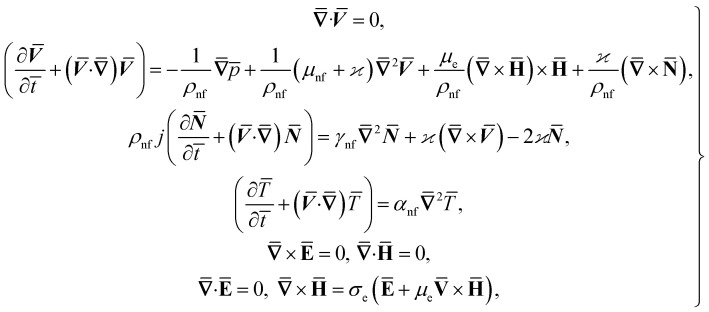
where **H̄**, and **Ē** are the magnetic and electric fields, respectively, in which ***H̄*** = *H*(cos *ϑe*_1_ + sin *ϑe*_2_) and 
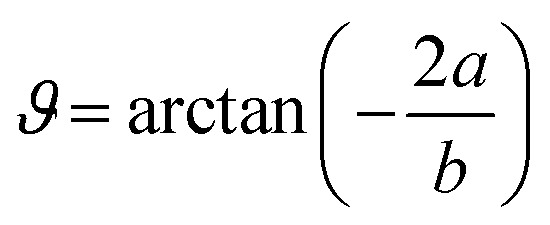
, **N̄** is the microrotation or angular velocity, *ρ*_nf_ is the density of nanofluid, nanofluids dynamic viscosity is *μ*_nf_, *ϰ* is the vortex viscosity, *j* is the microinertia coefficient, *γ*_nf_ is the spin-gradient viscosity, *μ*_e_ indicates the magnetic permeability, *σ*_e_ represent the electrical conductivity (*μ*_e_, *σ*_e_ = constants > 0).

For structure (1) we affix the boundary condition:2
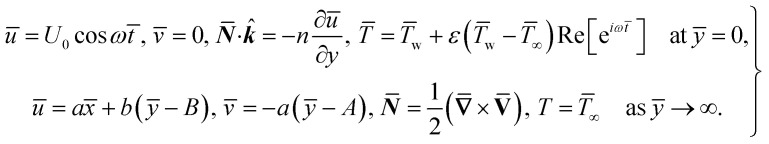
where *a* is the strength of an irrotational straining flow, *α* is a nondimensional constant which represent the ratio of the vorticity of a rotational shear flow to the strength of an irrotational straining flow, *A*, *B* are constants such that *A* is determined as part of the solution of the orthogonal flow, instead *B* is a free parameter. Also, *B* − *A* determines the displacement of the uniform shear flow parallel to the wall *y* = 0.

For the case of *n* = 0, we have ***N̄***·***k̂*** = 0 at the wall which shows strong concentration.^35^ Physically it means that microelements near the surface are unable to rotate.^36^ Further, for the case *n* = 1/2, narrates the disappearing of anti- symmetric portion of the stress tensor and indicates weak concentration^37^ of microelements. On the other side, at *n* = 1, flows indicate turbulent boundary layers.^38^

From conditions (2), mean that at infinity, 
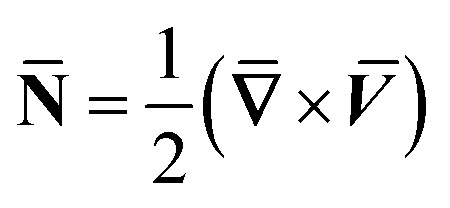
 shows that the micropolar fluid behaves like a classical fluid far away from the surface. Also, from free stream velocity, we can find that the stagnation point is 
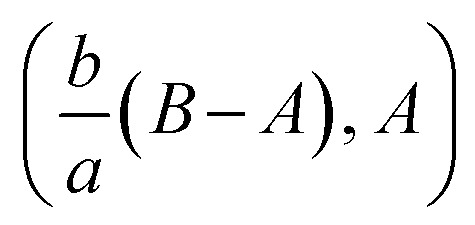
 and stream lines are hyperbolas whose asymptotes are:3
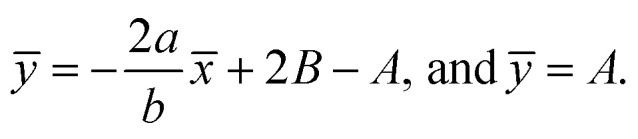


Considered the magnetic field **H̄** as defined in ref. 15 and assumed form of solutions as4
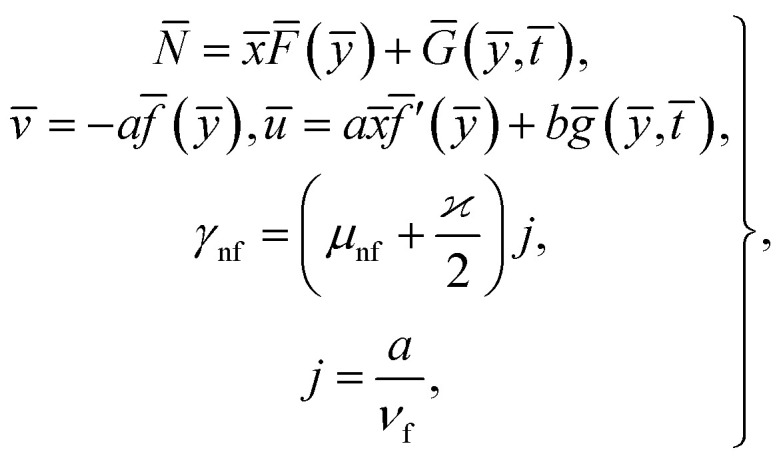


Eqn (1) and (2) takes the form5
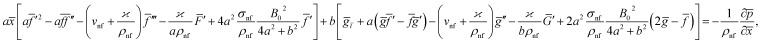
6
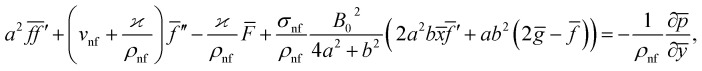
7
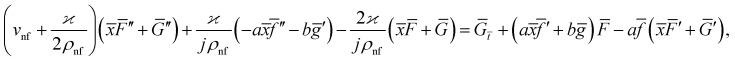
8
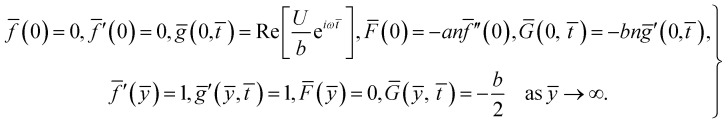


From (8), it is understood that the behavior of *f̄*(*ȳ*) and *ḡ*(*ȳ*, *t̄*) at infinity is

9 *f̄*(*ȳ*) ∼ *ȳ* − *A*, *ḡ*(*ȳ*, *t̄*) ∼ *ȳ* − *B*.

From eqn (7) and (8), we find the pressure field as^15^10
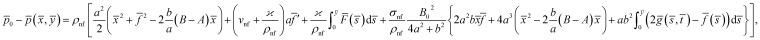
in which *p̄*_0_ is the stagnation pressure. From eqn (10), it is clearly seen that maximum pressure occurs at the stagnation point in through-out the flow domain.

Making use of eqn (10) and the following similarity solutions^15^11
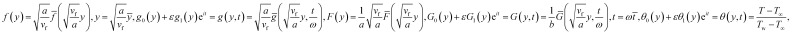
we obtain the flow field equations and the related boundary constraints, from eqn (5)–(9), in nondimensional form as12
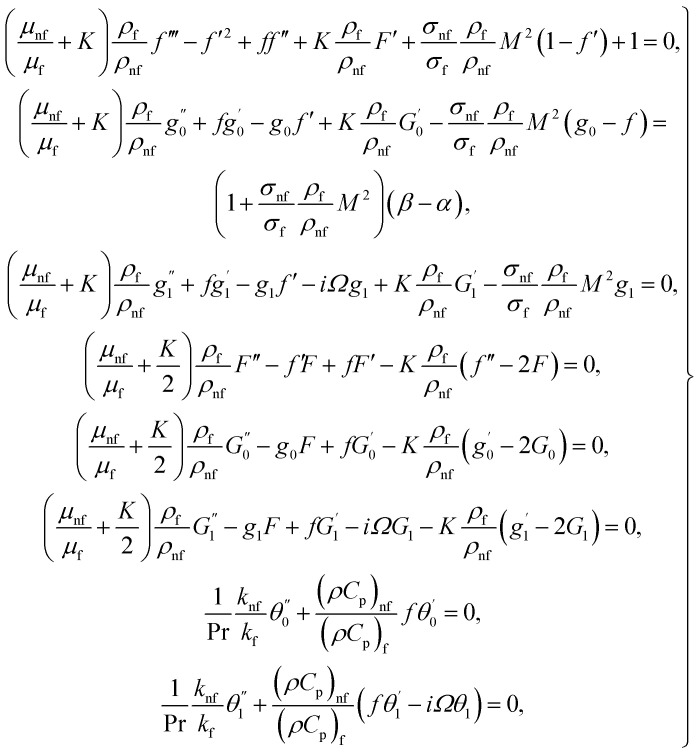
13
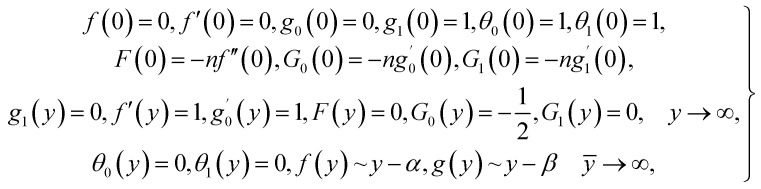
where
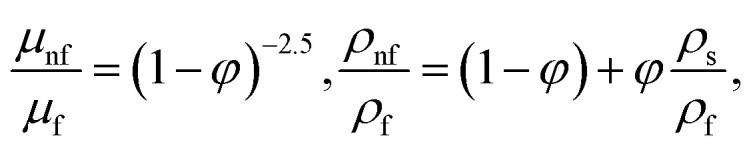

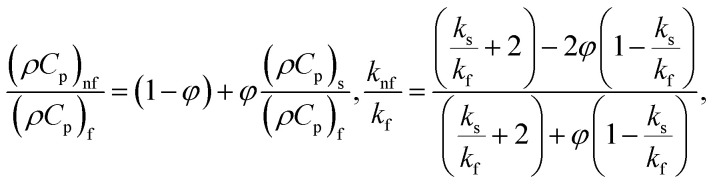

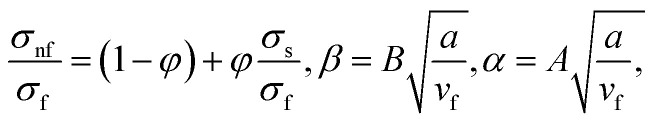

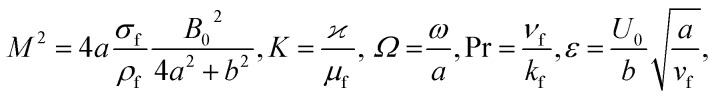
in which *φ* displays the volume fraction of nanoparticles, *ρ*_s_ and *ρ*_f_ are the density of solid fractions and base fluid, *k*_nf_ is the thermal conductivity of nanofluid, *k*_f_ and *k*_s_ are the thermal conductivity of base fluid the and solid fractions, respectively, (*ρC*_p_)_nf_ is the heat capacity of nanofluid, *K* is a material parameter, Pr is the Prandtl number, *M* is the Hartmann number, *ε* and *Ω* are the dimensionless amplitude and frequency of the wave.

The surface shear stress (*C*_f_) and heat transfer rate (Nu) in dimensionless form can be expressed as14
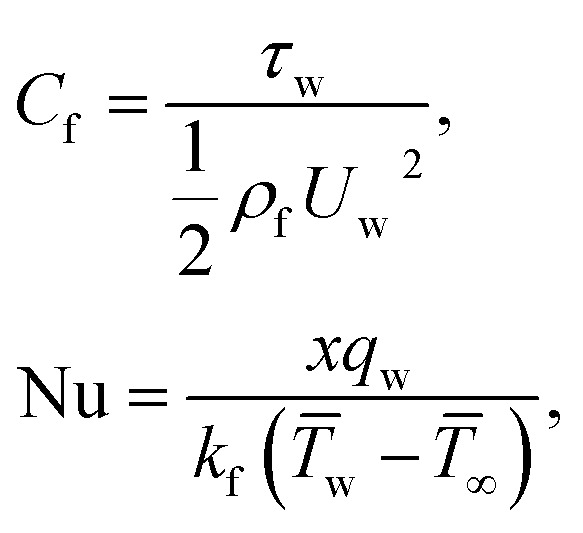
where *τ*_w_, is the wall shear stress and *q*_w_ the surface heat flux defines as15
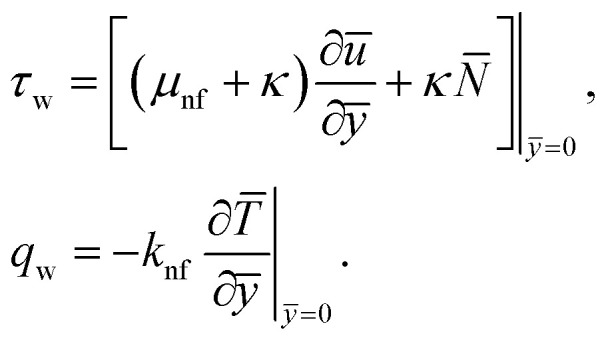


By using of (14), (17) and (18), we may write it as
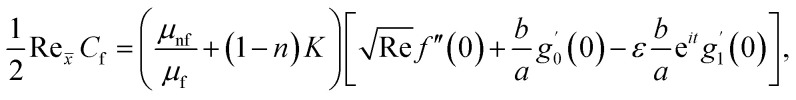

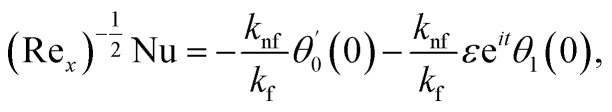
where Re_*x*_ = *ax*^2^/*v*_f_ is the local Reynolds number.

The equation of dividing streamline is16
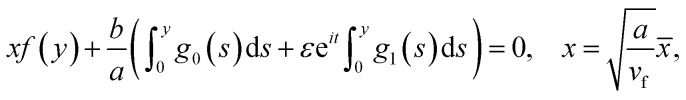
and meets the boundary *y* = 0.

Further, from eqn (12) and (18) we find the point of maximum pressure and point of zero skin friction as17
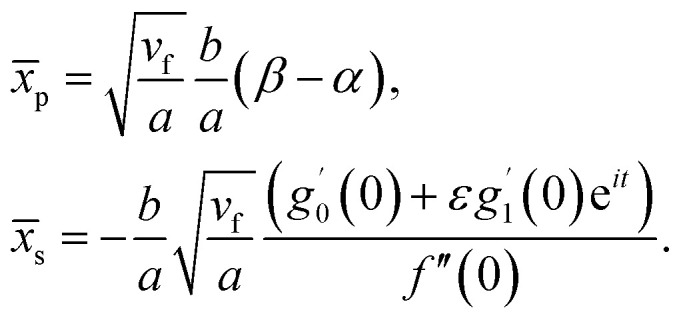


We note that *x̄*_p_ is independent of *M* whereas *x̄*_s_ depends on *M*. The ratio
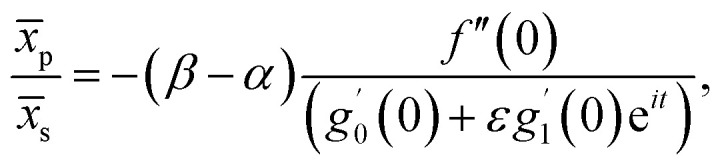
(for a fixed time) is identical for all angle of incidence.

## 3. Solution procedure

Numerical solution of *f*, *g*_0_, *F*, and *G*_0_-flow have been obtained numerically by means of midpoint method with Richardson extrapolation enhancement.

Furthermore, the series solutions of eqn (12)_3,6_ (*g*_1_(*y*) and *G*_1_(*y*) – flow) for small value of frequency *Ω* have been obtained as
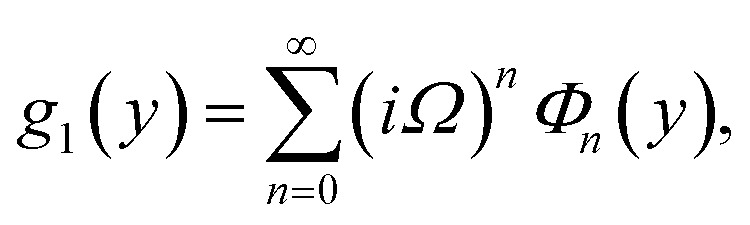
and
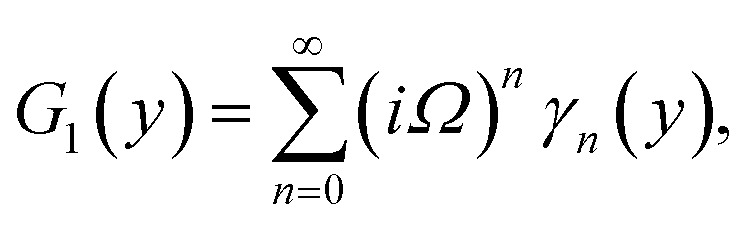


We are concerned only in real part of the solution. Thus*g*_1_(*y*) = *Φ*_0_(y) − *Ω*^2^*Φ*_2_(*y*) + *Ω*^4^*Φ*_4_(*y*)…where
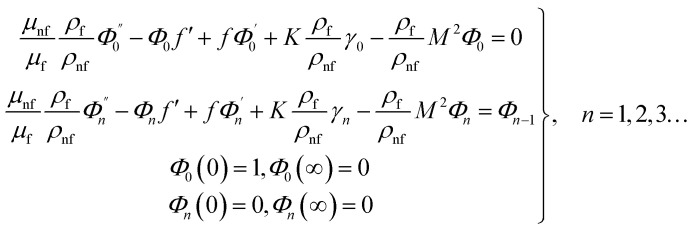


The above system has been tackled numerically using midpoint method with the Richardson extrapolation enhancement.

Similarly, for a small value of *Ω*, eqn (12)_8_ becomes
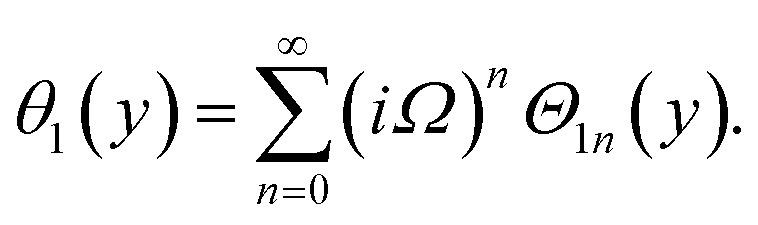
*θ*_1_(*y*) = *Θ*_10_(*y*) − *Ω*^2^*Θ*_12_(*y*) + *Ω*^4^*Θ*_14_(*y*)…

From (12)_7_ we have18
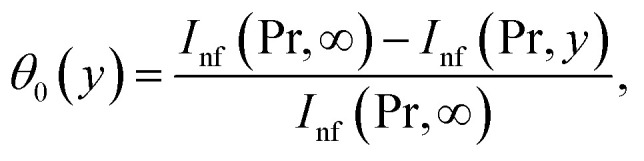
where
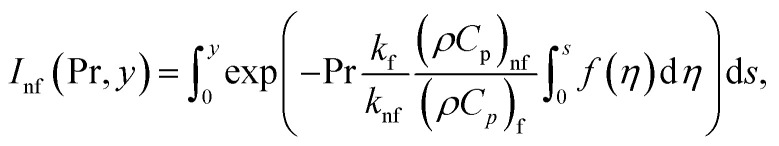
and
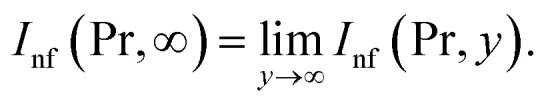


Making use of (12)_8_ we may write
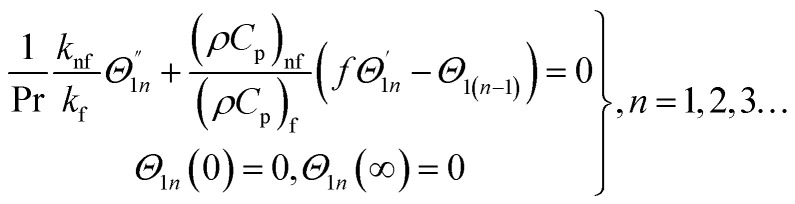
where *Θ*_10_(*y*) = *θ*_0_(*y*) is given in (18)

The numerical integration for the above system can be executed easily with aid of any mathematical software.

## 4. Results and discussion

Numerical assessment is carried out towards model equations of water-based micropolar nanofluid containing metals and oxide ceramics nanoparticles named as alumina (Al_2_O_3_) and copper (Cu). The range of solid volume fraction *φ* for the nanoparticles is maintained as 0 ≤ *φ* ≤ 0.2 along with the upper limit of Prandtl number 6.2 for base fluid *i.e.* water. Table 1 is used to present the thermos-physical properties of copper, alumina, and water. The numerical scheme is validated by constructing a discrete case of Hartmann number by ignoring the effects of nanoparticles shown in Table 2, we have found that our obtained results are agreed perfectly with.^40^

**Table 1:** Thermophysical characteristics of fluid and nanoparticles^15^

Thermophysical characteristics	*C*_p_ (J kg^−1^ K^−1^)	*ρ* (kg m^−3^)	*k* (W mK^−1^)	*σ* (S m^−1^)
Fluid phase (water)	4179	997.1	0.613	5.5 × 10^−6^
Copper (Cu)	385	8933	400	59.6 × 10^6^
Alumina (Al_2_O_3_)	765	3970	40	35 × 10^6^

**Table 2:** Comparison table for the values of *f*′′(0) when *K* = 0 (Newtonian fluid) and *φ* = 0 (base fluid)

***M***	*α*		*f*′′(0)	
	Present	39	Present	39
0	0.647901	0.6479	1.232588	1.2326
1	0.541007	0.5410	1.585331	1.5853
2	0.393589	0.3936	2.346663	2.3467
5	0.190729	0.1907	5.147964	5.1480
10	0.098774	0.0988	10.074741	10.0747

**Fig. 2:**
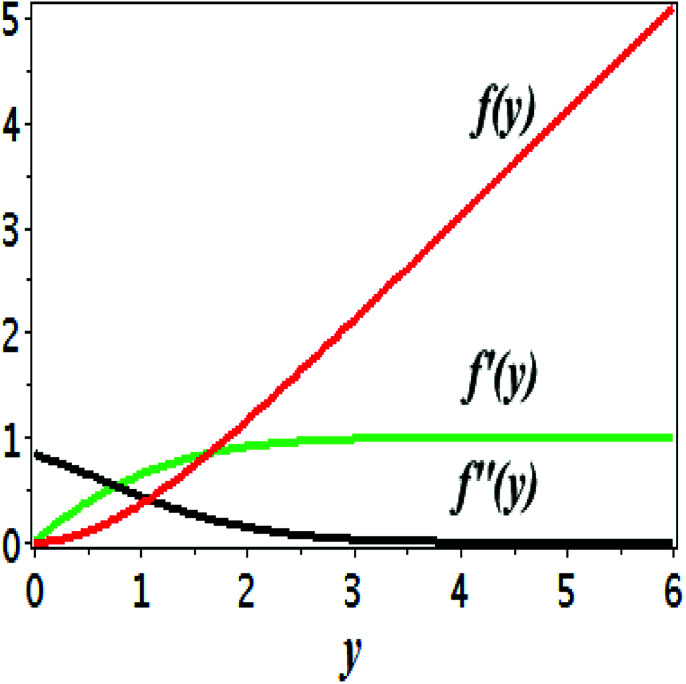
Plots showing *f*(*y*), *f*′(*y*), *f*′′(*y*) for *M* = 10^−7^, *φ* = 0.0, *K* = 0.0.

**Fig. 3:**
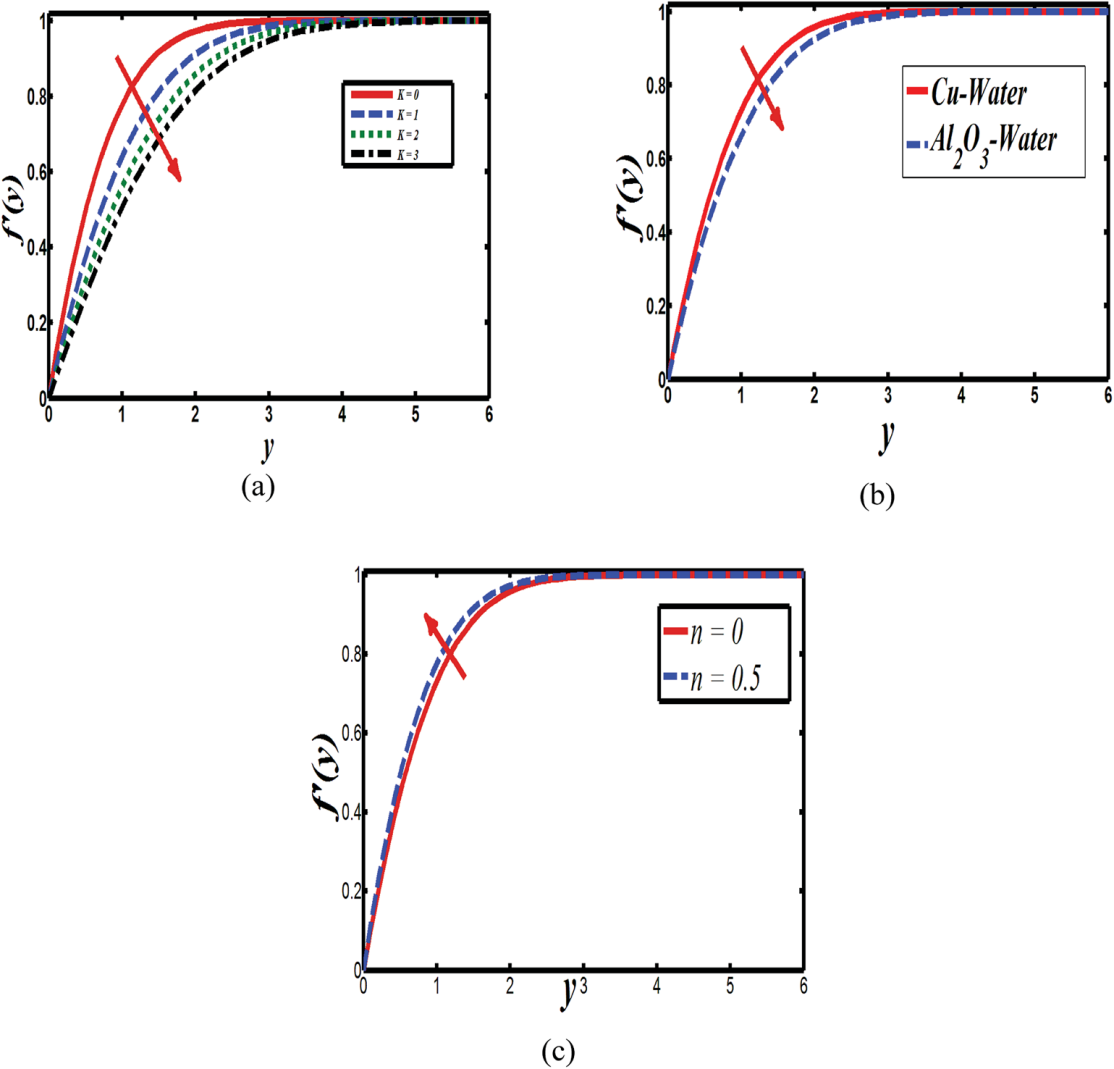
Plots showing *f*′(*y*) when *M* = 10^−7^ (a) Cu–water, *φ* = 0.0, *M* = 10^−7^, *n* = 0 (b) Cu–water, *n* = 0.1, *K* = 0.0 and (c) Cu–water, *K* = 0, *φ* = 0.0, *n* = 0, *K* = 0, *φ* = 0.1.

**Fig. 4:**
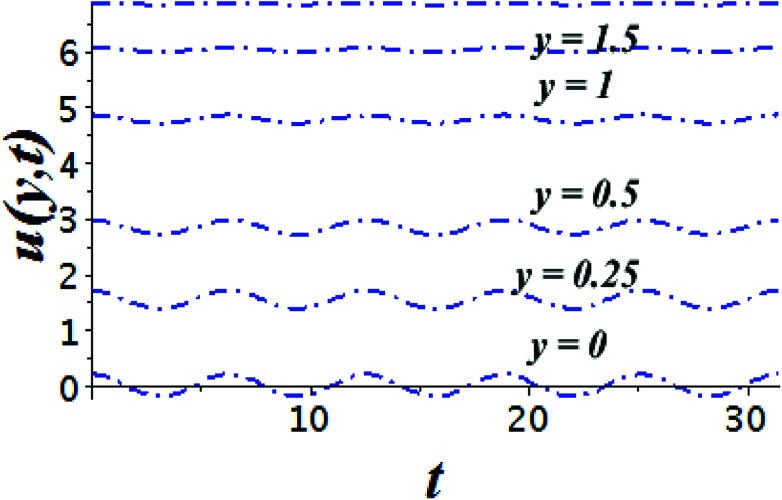
Time series of the flow of the velocity field *u*(*y*, *t*) at six different spaces from the surface for the time period *t* ∈ [0, 10π] for Cu–water, *φ* = 0.1, *K* = 0.0, *M* = 10^−7^, *ε* = 0.2, *Ω* = 0.2, *β* − *α* = −*α*, *x* = 1.

**Fig. 5:**
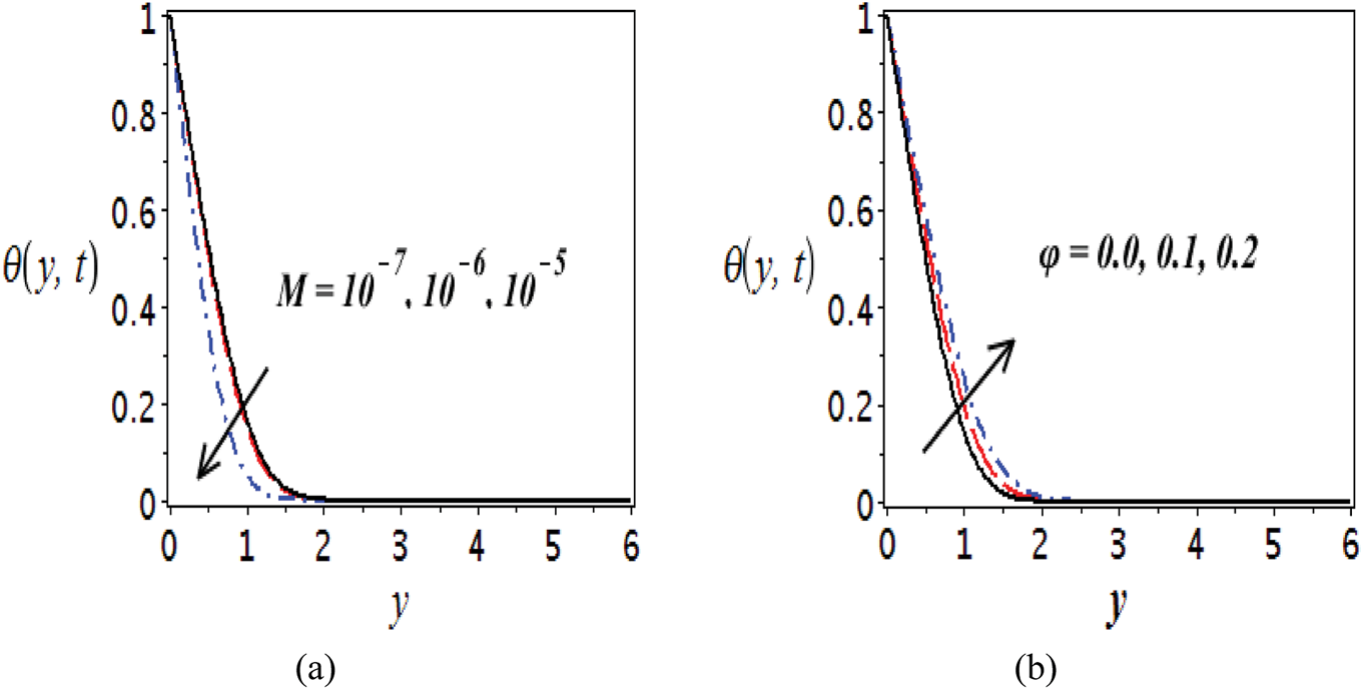
Plots presenting the variation of *θ*(*y*, *t*) for different parameters when Pr = 6.2, (a) Cu–water, *φ* = 0.0, *K* = 0.0 and (b) Cu–water, *M* = 10^−7^, *K* = 0.0.

**Fig. 6:**
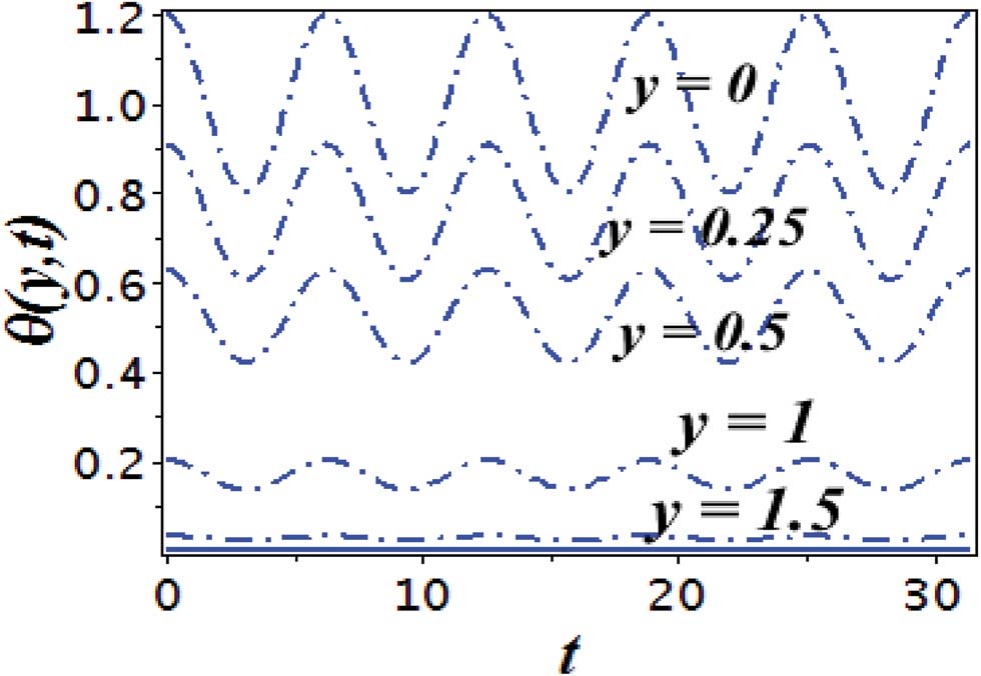
Time series of the flow of the temperature field *θ*(*y*, *t*)at five distinct spaces from the sheet for the time period *t* ∈ [0, 10π] for Cu–water, *φ* = 0.1, *K* = 0.0, *M* = 10^−7^, *ε* = 0.2, *Ω* = 0.2, *x* = 1.

**Fig. 7:**
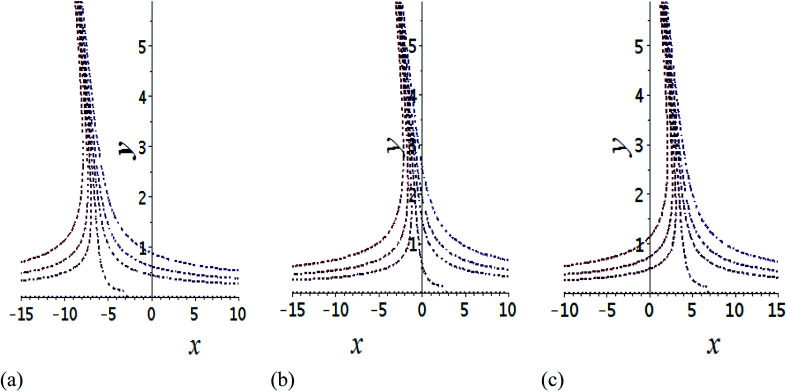
Shows the streamlines of Cu–water nanofluid when 
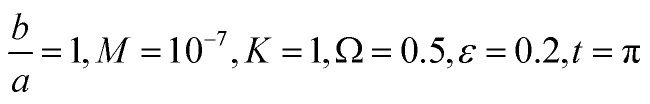
. (a) *β* − *α* = −5 − *α*, (b) *β* − *α* = 0, (c) *β* − *α* = 5 − *α*.

**Fig. 8:**
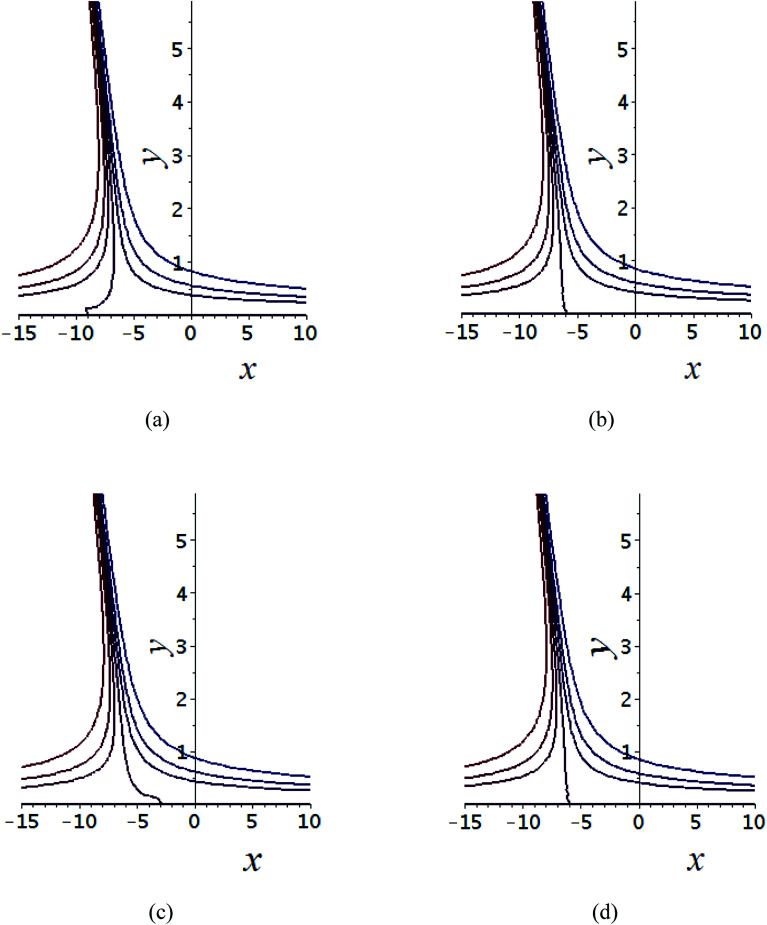
Shows the streamlines of Cu–water nanofluid when 
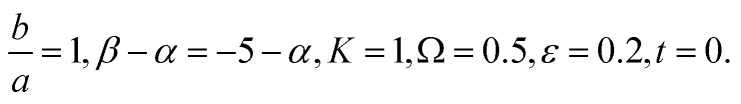
 (a) *t* = 0, (b) 
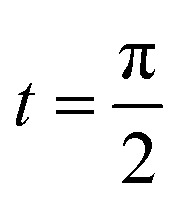
, (c) *t* = π, (d) 
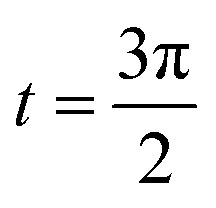
.

**Fig. 9:**
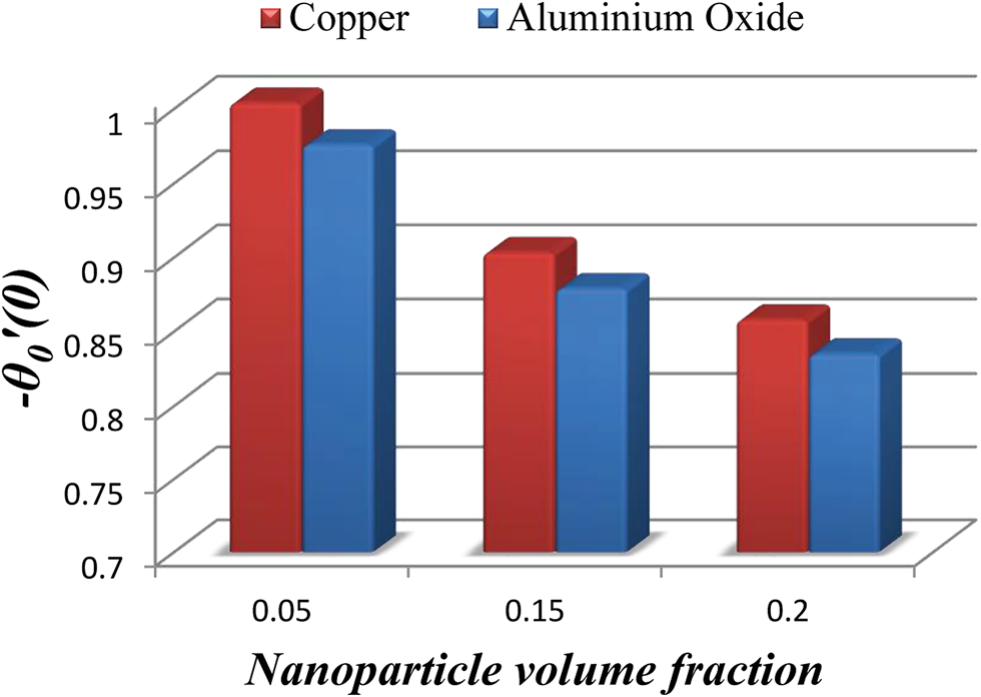
Bar graph evaluation of two nanoparticles concentrations 5%, 15% and 20% respectively.

The impact of relevant physical parameters on nanofluid velocity distributions is identified by Fig. 2–6. Fig. 2 shows the behavior of *f*(*y*), *f*′(*y*), *f*′′(*y*) for M = 10^−7^, *φ* = 0.0, *K* = 0.0. Fig. 3 is used to study the behavior of *f*′(*y*) towards unlike values of *K*, *φ*, *n* and different nanoparticles when base fluid is water. In Fig. 3(a), it is detected that momentum boundary layer thickness increases by growing the material parameter *K*. There is an important fact that Al_2_O_3_–water nanofluid produces a thicker velocity boundary layer than Cu–water as illustrated in Fig. 3(b). The strength of Fig. 3(c) is to draw out the impact of an imperative parameter *n*, the micro gyration parameter, which indicates the concentration of the micropolar fluid. From this figure, we tend to recognize that the velocity boundary layer thickness is thin just in case of week concentration as compared to strong concentration. Fig. 4 is designed in order to see the impact of time *t* on *u*. It is seen that *u* shows an oscillation performance with maximum amplitude at the surface and gradually declines away from the surface. Fig. 5 depicts the attitude of the temperature distribution *θ*(*y*, *t*) towards *φ* and *M* when Pr = 6.2. The influence of increasing Hartmann number on temperature profile, the decreasing nature of temperature field can be observed near the surface, while it shows a rise in behavior with the enhancement in nanoparticle volume fraction. The impact of time *t* on *θ*(*x*, *y*, *t*) is shown with the aid of Fig. 6. It is noticed that *θ*(*x*, *y*, *t*) exhibit waving nature and the amplitude of wave is found maximum near the surface and reduces far from the surface. Further, it is examined that the temperature is maximum at the surface, that is *y* = 0, and decrease away from it. The oblique flows are presented by way of streamline patterns in Fig. 7 and 8. The streamline come across the wall *y* = 0, at *x̄*_s_. It is concluded from these figures that their location is governed by *β* − *α* and time *t*. Fig. 9. shows the bar graph comparison of both copper and aluminium oxide nanoparticles. It demonstrates that copper has a higher surface temperature gradient when contrasted with the aluminium oxide nanoparticles. As Cu has the highest value of thermal conductivity as compared to TiO_2_ and Al_2_O_3_. The reduced value of thermal diffusivity leads to higher temperature gradients and, hence, higher improvements in heat transfer. More real applications of nanofluids include different types of microchannels, heat exchangers, thermosyphons, heat pipes, chillers, car radiators, cooling and heating in buildings, solar collectors, air conditioning and refrigeration, cooling of electronics, in diesel electric generator as jacket water coolant, nanofluids in transformer cooling oil, in drag reductions and many others.

Tables 3–10 delineate the impacts of the involved parameter on the physical quantities near the wall for both copper and aluminium oxide nanoparticles when water is preserved as a base fluid. We comment that the estimations of *α* and *f*′′(0) rely on upon *M*, *φ* and, *K*, as should be obvious from Tables 3 and 4. More precisely, *f*′′(0) increases and *α* decreases as *φ* and *M* are increases. Moreover, increases in material parameter *K* cause an increase in *α* and decrease in *f*′′(0). Tables 5 and 6 shows the numerical values of velocity gradient at the surface against *M*, *φ*, *K* and *β* − *α* = −*α*, 0, *α* and it is noticed that the magnitude of *g*_0_(*y*) does not depend on *φ*. As far as the variation of *g*_0_(*y*) against M and K are concerned, is found its magnitude shows increments when *M* rises while shows decline nature for all *β* − *α* when *K* increases. The rapid increase is found for Cu–water nanofluid as compared to Al_2_O_3_–water. The numerical variation of 
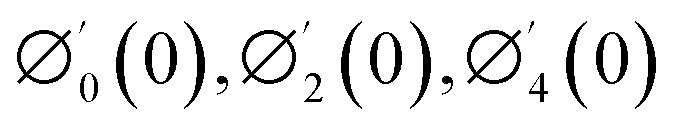
 against *M*, *φ*, and *K* are revealed in Tables 7 and 8. Generally, magnetohydrodynamic (MHD) flow plays the main role in the manufactured products and several businesses like pumps and oil purification, *etc.* In Tables 9 and 10, it is found that the gradient of temperature is decreasing function of both material parameter *K* and nanoparticle volume fraction *φ*. Thus, the rate of heat transfer increase near the surface. It is important to note that Cu–water remarks higher heat transfer rate as compared to Al_2_O_3_–water nanofluid. As Cu has the highest value of thermal conductivity as compared to Al_2_O_3_. The reduced value of thermal diffusivity leads to higher temperature gradients and, hence, higher improvements in heat transfer. Furthermore, it is also noticed that the temperature gradient shows inciting attitude when we increase Hartmann number *M* which brings enhancement in heat transfer rate near the surface. In general, micropolar fluids deal excessive resistance to the fluid motion rather than the Newtonian fluid. This occurrence also demonstrates that the greater micropolar parameter improves the total viscosity in the fluid flow. Thus, the micropolar fluid is a very effective fluid medium in the boundary layer for observing the laminar flow.

**Table 3:** Tabular value of *α* and *f*′′(0) for *K*, *M*, *φ* and *n*

Cu–water						
*M*	*K*	*φ*	*n* = 0.0		*n* = 0.5	
			*α*	*f*′′(0)	*α*	*f*′′(0)
10^−7^	1	0.1	0.722568	1.069269	0.647978	1.233009
10^−6^			0.644688	1.264293	0.573809	1.440364
10^−5^			0.142575	6.935249	0.116581	7.227920
10^−7^	2		0.848297	0.868343	0.732395	1.09084
	3		0.950844	0.742678	0.808043	0.988713
	4		1.039219	0.655790	0.877179	0.910772
	1	0.05	0.782726	0.972330	0.696955	1.140483
		0.15	0.680741	1.139190	0.615133	1.293535
		0.2	0.657732	1.185850	0.599389	1.327287

**Table 4:** Numerical value of *α* and *f*′′(0) for *K*, *M*, *φ* and different *n*

Al_2_O_3_–water						
*M*	*K*	*φ*	*n* = 0.0		*n* = 0.5	
			*α*	*f*′′(0)	*α*	*f*′′(0)
10^−7^	1	0.1	0.846878	0.903951	0.762347	1.047950
10^−6^			0.770550	1.040532	0.689359	1.194029
10^−5^			0.185034	5.324346	0.152298	5.584560
10^−7^	2		0.990380	0.730675	0.861656	0.927134
	3		1.107115	0.623266	0.950585	0.840333
	4		1.207568	0.549524	1.031693	0.774098
	1	0.05	0.861087	0.877879	0.769082	1.033635
		0.15	0.835022	0.919714	0.756813	1.050349
		0.2	0.833913	0.925333	0.753188	1.061526

**Table 5:** Numerical value of *β* − *α* and 
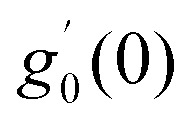
 for *K*, *M*, *φ* and different *n*

Cu–water						
*M*	*K*	*φ*	*n* = 0.0		*n* = 0.5	
			*β* − *α*	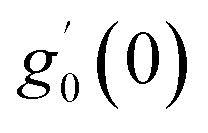	*β* − *α*	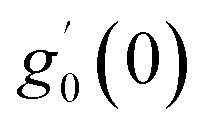
10^−7^	1	0.1	−0.722568	1.271410	−0.647978	1.406550
			0	0.498790	0	0.607587
			0.722568	−0.273830	0.647978	−0.191375
10^−6^			−0.644688	1.294936	−0.573809	1.408199
			0	0.479861	0	0.581705
			0.644688	−0.335214	0.573809	−0.244789
10^−5^			−0.142575	1.459372	−0.116581	1.366378
			0	0.470574	0	0.523735
			0.142575	0.518223	0.116581	−0.318907
10^−7^	2		−0.848297	1.185937	−0.732395	1.406516
			0	0.449325	0	0.607585
			0.848297	−0.287287	0.732395	−0.191346
	3		−0.950844	1.126700	−0.808043	1.406519
			0	0.420537	0	0.607598
			0.950844	−0.285624	0.808043	−0.191322
	4		−1.039219	1.083121	−0.877179	1.406571
			0	0.401646	0	0.607674
			1.039219	−0.279829	0.877179	−0.191222
	1	0.05	−0.782726	1.253428	−0.696955	1.406544
			0	0.489102	0	0.607715
			0.782726	−0.275223	0.696955	−0.191113
		0.15	−0.680741	1.253428	−0.615133	1.406544
			0	0.489102	0	0.607715
			0.680741	−0.275223	0.615133	−0.191113
		0.2	−0.657732	1.253428	−0.599389	1.406544
			0	0.489102	0	0.607715
			0.657732	−0.275223	0.599389	−0.191113

**Table 6:** Numerical value of *β* − *α* and 
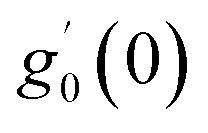
 for *K*, *M*, *φ* and different *n*

Al_2_O_3_–water						
*M*	*K*	*φ*	*n* = 0.0		*n* = 0.5	
			*β* − *α*	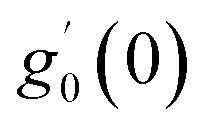	*β* − *α*	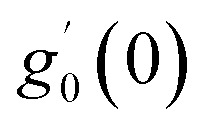
10^−7^	1	0.1	−0.838110	1.263658	−0.754251	1.406554
			0	0.497639	0	0.607660
			0.838110	−0.268379	0.754251	−0.191234
10^−6^			−0.763937	1.282825	−0.683338	1.407946
			0	0.481221	0	0.585292
			0.763937	−0.320383	0.683338	−0.237360
10^−5^			−0.184939	1.450722	−0.152254	1.371608
			0	0.465719	0	0.520896
			0.184939	−0.519283	0.152254	−0.329814
10^−7^	2		−0.980384	1.171577	−0.852508	1.406571
			0	0.447083	0	0.607713
			0.980384	−0.277409	0.852508	−0.191145
	3		−1.096144	1.109010	−0.940504	1.406727
			0	0.418007	0	0.607960
			1.096144	−0.272996	0.940504	−0.190805
	4		−1.195780	1.063927	−1.020783	1.407090
			0	0.399428	0	0.608598
			1.195780	−0.265071	1.020783	−0.189894
	1	0.05	−0.861087	1.247827	−0.769082	1.406550
			0	0.488260	0	0.607789
			0.861087	−0.271306	0.769082	−0.190972
		0.15	−0.835022	1.247827	−0.756813	1.406550
			0	0.488260	0	0.607789
			0.835022	−0.271306	0.756813	−0.190972
		0.2	−0.833913	1.247827	−0.753188	1.406550
			0	0.488260	0	0.607789
			0.833913	−0.271306	0.753188	−0.190972

**Table 7:** Variation 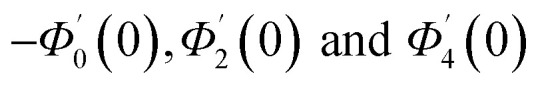 for *N*_1_, *M*, *φ* and distinct *n*

Cu–water								
*M*	*K*	*φ*	*n* = 0.0			*n* = 0.5		
			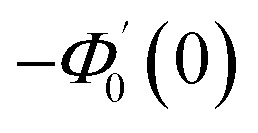	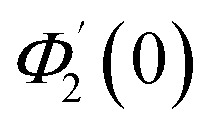	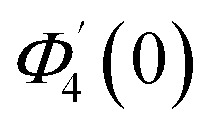	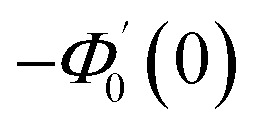	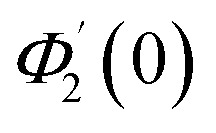	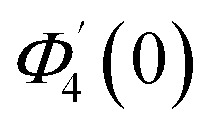
10^−7^	1	0.1	0.691779	0.085912	0.010277	0.813191	0.093913	0.011531
10^−6^			0.945615	0.050471	0.003659	1.083458	0.055256	0.003881
10^−5^			6.877988	0.000465	0.000018	7.116813	0.010491	0.000744
10^−7^	2		0.551502	0.069872	0.007756	0.719432	0.083075	0.010198
	3		0.466232	0.058929	0.006346	0.652080	0.075288	0.009240
	4		0.408703	0.051265	0.005471	0.600686	0.069334	0.008499
	1	0.05	0.625838	0.078389	0.009190	0.751624	0.087123	0.010723
		0.15	0.625838	0.078389	0.009190	0.751624	0.087123	0.010723
		0.2	0.625838	0.078389	0.009190	0.751624	0.087123	0.010723

**Table 8:** Behavior of 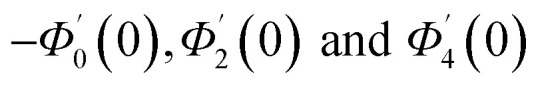 for *N*_1_, *M*, *φ* and *n*

Al_2_O_3_–water								
*M*	*K*	*φ*	*n* = 0.0			*n* = 0.5		
			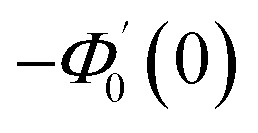	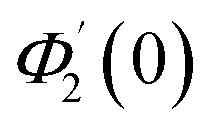	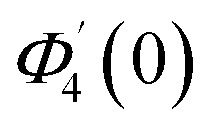	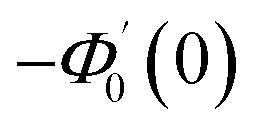	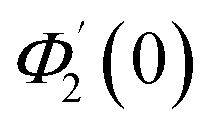	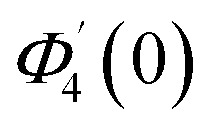
10^−7^	1	0.1	0.589384	0.073201	0.008584	0.698279	0.080806	0.009934
10^−6^			0.766655	0.047268	0.003592	0.888616	0.051682	0.003936
10^−5^			5.270802	0.000533	0.000015	5.491650	0.006956	0.000369
10^−7^	2		0.467838	0.058713	0.006477	0.617781	0.071474	0.008779
	3		0.394924	0.049220	0.005321	0.559961	0.064732	0.007922
	4		0.346119	0.042662	0.004559	0.515870	0.059506	0.007218
	1	0.05	0.567381	0.071016	0.008240	0.685102	0.079566	0.009805
		0.15	0.567381	0.071016	0.008240	0.685102	0.079566	0.009805
		0.2	0.567381	0.071016	0.008240	0.685102	0.079566	0.009805

**Table 9:** Variation of 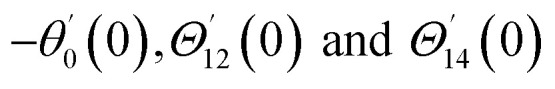 for *K*, *M*, *φ* and *n* with Pr = 6.2

Cu–water								
*M*	*K*	*φ*	*n* = 0.0			*n* = 0.5		
			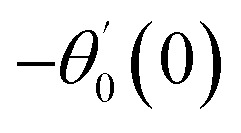	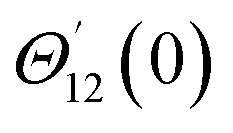	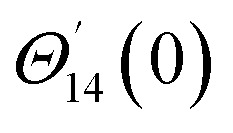	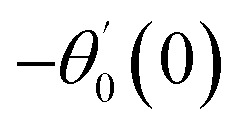	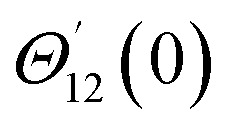	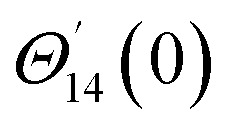
10^−7^	1	0.1	0.976002	0.625531	0.894378	1.010236	0.511496	0.817287
10^−6^			1.014206	0.502684	0.810538	1.047785	0.415034	0.743960
10^−5^			1.416020	0.094820	0.374691	1.434746	0.087727	0.361806
10^−7^	2		0.926816	0.852550	1.026503	0.976897	0.628893	0.895597
	3		0.891638	1.077594	1.138896	0.950471	0.746466	0.965929
	4		0.864509	1.301745	1.238182	0.928661	0.864375	1.030293
	1	0.05	1.003951	0.899119	1.094526	1.043860	0.713781	0.987421
		0.15	0.903354	0.596328	0.841090	0.938019	0.479655	0.762802
		0.2	0.857919	0.490484	0.741267	0.890235	0.397167	0.674087

**Table 10:** Tabular form of 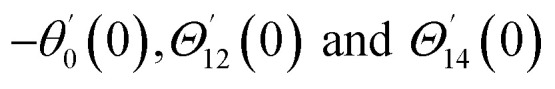 for *K*, *M*, *φ* and *n* when Pr = 6.2

Al_2_O_3_–water								
*M*	*K*	*φ*	*n* = 0.0			*n* = 0.5		
			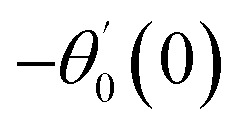	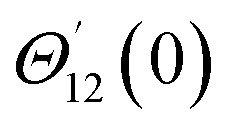	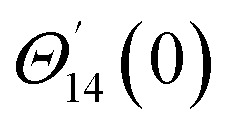	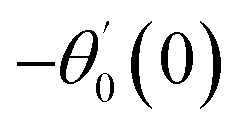	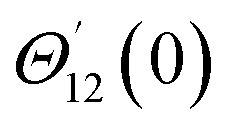	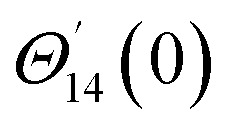
10^−7^	1	0.1	0.932422	0.834821	1.016303	0.966518	0.675214	0.924473
10^−6^			0.964089	0.686724	0.931296	0.997735	0.559994	0.850331
10^−5^			1.360115	0.111887	0.405929	1.380663	0.102134	0.389729
10^−7^	2		0.884116	1.152682	1.172595	0.933797	0.838537	1.017006
	3		0.849870	1.468078	1.304933	0.907950	1.002624	1.100002
	4		0.823621	1.782222	1.421429	0.886673	1.167569	1.175876
	1	0.05	0.976038	1.077483	1.185520	1.015805	0.849488	1.066400
		0.15	0.878960	0.709963	0.908840	0.913561	0.567020	0.821797
		0.2	0.835014	0.581655	0.799721	0.867296	0.467622	0.725061

## 5. Concluding remarks

The properties of magneto-hydrodynamic oblique stagnation point flow of micropolar nanofluid over an oscillatory plate were reported by way of parametric study. In this attempt, we have chosen alumina Al_2_O_3_ and copper Cu as nanoparticles when water is treated as base a fluid. The key finding of the current analysis is itemized as follows

• The momentum boundary layer is thicker for Al_2_O_3_–water as associated to Cu–water. In addition, Al_2_O_3_–water results show more surface temperature while Cu–water generates the lowest surface temperature.

• Thickness decay is found for momentum boundary layer against increasing value of nanoparticles volume fraction while opposite attitude towards material parameter. Further, the thickness of momentum boundary layer is thin for the case of weak concentration as compared to strong concentration.

• The local wall shear stress is the increasing function of Hartmann number and material parameter while it shows opposite attitude towards nanoparticles volume fraction. It was also noticed that Cu–water with the comparison of Al_2_O_3_–water gives maximum local wall shear stress.

• The magnitude of rate of heat transfer is significantly large for Cu–water as compared to Al_2_O_3_–water. On the other hand, the heat transfer rate near the plate surface is declining function of Hartmann number while the contrary trend is found for both material parameter and nanoparticles volume fraction.

## Conflicts of interest

There are no conflicts of interest to declare

## Supplementary Material
